# The Impact of Non-Acoustic Factors on Chinese Community Response to Noise: A Systematic Review

**DOI:** 10.3390/ijerph22040651

**Published:** 2025-04-21

**Authors:** Wenkai Wang, Hui Ma, Chao Wang

**Affiliations:** School of Architecture, Tianjin University, Weijin Road 92, Nankai District, Tianjin 300072, China; wangwk2001@tju.edu.cn (W.W.); pdwangchao@tju.edu.cn (C.W.)

**Keywords:** non-acoustic factors, noise annoyance, community response to noise

## Abstract

Noise pollution has become one of the most prominent environmental issues in China. Although many studies have summarized the impact of non-acoustic factors on noise annoyance, the unique mechanisms of these factors within the context of Chinese society and culture still require systematic investigation. In this study, a systematic review of articles obtained from the CNKI, WanFang, WoS Core Collection, and Scopus databases (up to December 2024) was conducted, and 42 articles were included in a qualitative analysis to summarize the patterns of non-acoustic factors’ influence on the community response of Chinese residents to noise. The results revealed the following: (1) The effects of non-acoustic factors on Chinese residents are significant, with the trends for factors such as noise sensitivity, attitude to noise source, health status, perceived quality of the living environment, and education level influencing the Chinese community response to noise having been basically clarified. However, the influence of the remaining factors and the unique influences of various non-acoustic factors await further quantitative analyses. (2) Interactions among various factors deserve close attention. The interactions between non-acoustic factors, as well as those between non-acoustic factors and sound source types, have been reflected in some studies. These may be significant for explaining the effects of non-acoustic factors and merit further research. (3) Compared to international studies, research on non-acoustic factors in China is relatively limited in quantity and unevenly distributed, which is insufficient to support further quantitative analysis or a detailed exploration of the underlying mechanisms. Therefore, more studies are necessary to support the future rationalization of noise policies and national standards in China.

## 1. Introduction

According to the Annual Report on Prevention and Control of Noise Pollution in China, noise disturbance issues accounted for 61.3% of all ecological and environmental pollution reports in 2023, ranking first among all environmental pollution factors [1]. This indicates that noise problems have become a major environmental issue affecting Chinese residents, reflecting the current severe noise pollution situation in China.

The impacts of noise pollution on human health include sleep disorders [2], attention and cognitive impairments [3], and adverse psychological responses (e.g., annoyance) [4]. The long-term cumulative effects of noise exposure may also induce various diseases, such as hypertension and coronary heart disease [5,6,7]. In order to investigate the mechanisms of noise impact, various indicators have been used to measure the effects of noise [8,9,10]. Among these indicators, noise annoyance is widely recognized as an important indicator of community response and has been extensively applied in the evaluation and study of noise pollution. Studies have confirmed that acoustic factors—such as noise exposure levels [11], frequency [12,13], and the number of sound sources [14]—have significant effects on noise annoyance. However, various non-acoustic factors, such as noise sensitivity, age, and housing factors, also influence noise annoyance [15]. For example, individuals with high noise sensitivity tend to experience greater annoyance caused by noise [16]. In certain cases, non-acoustic factors can even exert a more pronounced effect [17].

To systematically review the influence of non-acoustic factors on noise annoyance, Nelson [15] summarized the non-acoustic factors affecting transportation noise, proposing two main categories—attitudinal factors and demographic factors—and examined their roles in the process of noise perception. Additionally, Fields [16] reviewed the influence of personal and situational factors on noise annoyance. On this basis, the categorization of non-acoustic factors has been continuously expanded and updated [18,19], rendering it more comprehensive and systematic. In order to quantify the specific effects of non-acoustic factors on noise annoyance, Miedema and Vos [20] integrated data from multiple studies to conduct a quantitative analysis of the influence of various non-acoustic factors, reaching several classic conclusions that underscore the significant role of non-acoustic factors in noise perception.

Multiple studies have confirmed that different cultural backgrounds can lead to various noise responses [21,22,23]. Therefore, whether the conclusions of the aforementioned research are consistent with the responses of Chinese residents remains in question. With the continuous development of research on China’s acoustic environment, a certain body of studies on the effects of noise has been accumulated. Thus, synthesizing a similar body of studies for Chinese residents is both feasible and necessary. Accordingly, the present study systematically reviews and analyzes the literature focusing on Chinese residents, summarizing the current status and trends of research on non-acoustic factors, with particular attention paid to their underlying mechanisms. The findings of this study will fill a research gap and facilitate the development of a noise impact assessment system that better reflects China’s socio-cultural characteristics, providing a more precise basis for environmental noise management and policy-making.

## 2. Methods

### 2.1. Search Strategy

This systematic review followed the Prisma Statement [24]. Since this study is a qualitative review rather than strictly a summary of all research data, it was not registered nor prepared following a review protocol.

The literature search was conducted on 6 December 2024, across four major online databases: CNKI, WanFang, WoS Core Collection, and Scopus. To comprehensively cover the relevant research, the search keywords were set as “noise” AND “annoyance” and limited to institutions in China, with no language restrictions. The same retrieval method was used to retrieve research conducted by non-Chinese institutions but focusing on Chinese residents.

### 2.2. Exclusion and Inclusion Criteria

The initial search yielded 894 articles, and after removing duplicates, 640 articles were screened. The inclusion criteria applied at this stage were as follows: (1) articles involving individuals’ subjective perceptions of the noise environment; (2) articles incorporating non-acoustic factors into their analyses (excluding research focusing solely on sensory factors unrelated to noise sources, such as color or odor); and (3) articles set in China. In total, 88 articles were selected for full-text assessment.

The inclusion criteria for full-text assessment were as follows: (1) research that clearly identified noise annoyance as a core indicator and used standardized scales; (2) research that explicitly examined the relationship between non-acoustic factors and noise annoyance and provided a statistical analysis. After a rigorous screening process, 47 articles that failed to meet the inclusion criteria were excluded, leaving 41 qualified studies. In addition, one relevant study conducted by a non-Chinese institution but focusing on Chinese residents was included, resulting in a final total of 42 studies for analysis.

**Figure 1:**
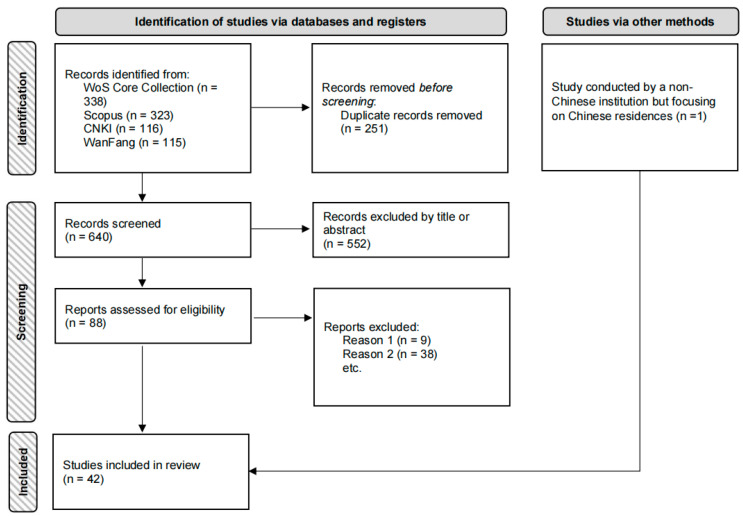
A flow chart showing article selection, screening, and exclusion in this systematic review.

The above process was conducted by two researchers who independently reviewed the articles, with any discrepancies discussed and resolved by two senior researchers in the field. The process of article selection, screening, and exclusion for this systematic review is shown in Figure 1.

## 3. Results

### 3.1. Overall Research Trends

In general, there has been an increasing focus on non-acoustic factors in China in recent years, but the relevant research in China is relatively limited in quantity and unevenly distributed across different non-acoustic factors.

**Figure 2:**
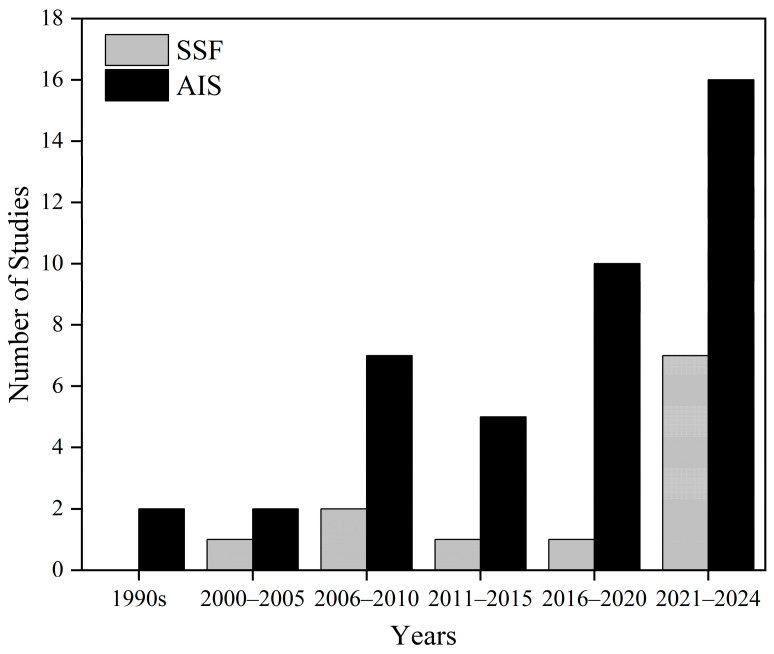
Yearly distribution of included studies. Note: SSF refers to studies specifically focusing on the impact of non-acoustic factors on noise annoyance, and AIS refers to all included studies.

Figure 2 shows that the number of studies regarding non-acoustic factors in China has shown an upward trend, and that studies specifically focusing on the mechanisms of non-acoustic factors have emerged in recent years.

In terms of the number of non-acoustic factors, a total of 20 were analyzed in the included studies. Referring to the conventional method [18,19,20], these factors can be classified into attitudinal, demographic, and situational factors (Table 1). Ranked by research frequency, the top five factors were gender, age, noise sensitivity, educational level, and occupation, while most of the remaining factors were explored fewer than five times. Compared with international research, the discussion on housing-related factors is still lacking in China. Moreover, except for age, the effects of other factors on the Chinese community response have not been summarized or analyzed, whereas international studies [18,20] have already summarized the impacts of noise sensitivity, fear, and most demographic factors.

### 3.2. Effects of Non-Acoustic Factors

The effects of each non-acoustic factor will be compared with those in international research [18,20] in order to determine the influence of non-acoustic factors in the Chinese context.

#### 3.2.1. The Impact of Attitudinal Factors on Noise Annoyance

After Nelson’s comprehensive summary [15], the components of attitudinal factors have been largely clarified. In this study, the attitudinal factors are categorized into five dimensions for discussion: noise sensitivity, attitude to noise source, perceived quality of the neighborhood, activity during exposure, and fear.

**Figure 3:**
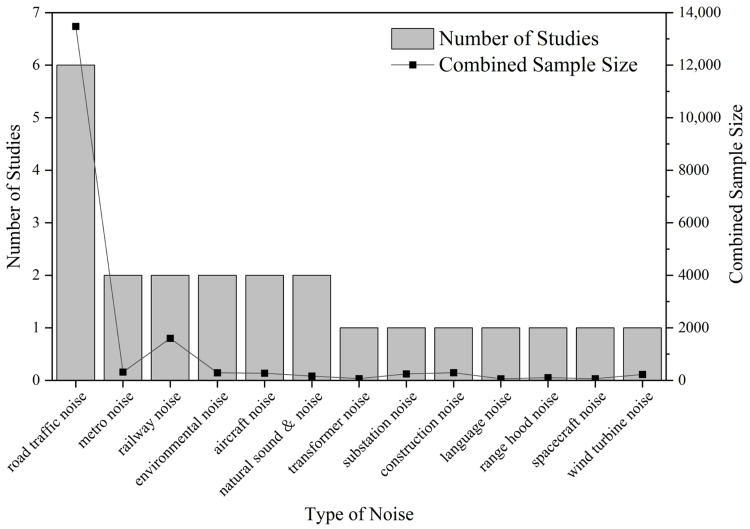
Distribution of noise types and sample sizes in noise sensitivity studies in China.

Noise sensitivity is the most frequently studied attitudinal factor in China, having been examined with a particular focus on transportation noise (Figure 3). In general, Chinese research shows a similar trend to international ones: those with higher noise sensitivity will be more annoyed. However, only a few studies have quantified its effect: Di et al. found that highly sensitive individuals scored 1.4 points higher on annoyance compared with low-sensitivity individuals (using an 11-point numeric scale) [25], while Zheng et al. reported that the odds of annoyance increased 3.08-fold per one-unit increase in noise sensitivity [26]. Moreover, the effect of noise sensitivity varies across different noise types. For instance, the difference in annoyance level between high- and low-sensitivity individuals differs between speech and road traffic noise [27], and the influence pathways of noise sensitivity also differ by noise source [28].

Since the available data remain limited, the effects of noise sensitivity are currently difficult to quantitatively compare with those in international studies. Additionally, distinguishing the effect of noise sensitivity across different noise types remains a challenge.

The attitude to the noise source also has an impact on the Chinese community response to noise, with this factor generally having been defined from two perspectives in the reviewed research (Table 2): (1) a comprehensive consideration of the impact of noise sources, including social value, convenience, safety, etc. [28,29,30,31]; and (2) a focus solely on evaluating a single impact of the noise [32,33]. In contrast, international scholars have additionally focused on the possibility of residents protecting themselves from noise [20]. Despite the inconsistency in definition, a consistent conclusion emerges: individuals with a negative attitude toward noise or noise sources experience higher levels of annoyance. Research indicates that such an attitude can be modified: Lam et al. [31,34] found that implementing positive noise control measures could encourage more favorable attitudes among residents, but this effect is not long-lasting. Similarly, international research [20] pointed out that economic dependence on noise sources, people’s engagement in activities related to those sources, and the regular use of noise sources can influence individuals’ attitudes toward noise. These findings offer insights for noise policy development: noise annoyance can be reduced by combining short-term informational interventions (e.g., promoting positive noise control measures) with long-term benefit-building strategies (e.g., encouraging residents to use public transportation). Furthermore, the consistent trends in influence allow for simplified descriptions based on practical needs.

The perceived quality of the living environment frequently influences noise annoyance. Several studies have confirmed that a higher perceived quality of the living environment leads to lower levels of noise annoyance [23,29,35]. However, Lam et al. found that this factor had little or no effect on the perception of railway traffic noise [28,31], which contrasts with the conclusions of Zhang et al. [29]. Due to the limited number of relevant studies, the specific factors leading to this discrepancy remain unclear.

Activity during exposure can lead to different perceptions of annoyance. Liu found different exposure–annoyance relationships according to activity type [36]. Similarly, Zhang et al. [37,38] observed that using different experimental tasks in their research resulted in varying levels of annoyance.

The role of fear has only been mentioned in one study: Qu et al. confirmed that fear of flying increased noise annoyance [39], but no quantitative analysis was conducted. According to Miedema and Vos [20], fear could have an impact equivalent to up to 19 dB DNL. Nelson suggested that fear arises from the potential danger posed by traffic activities [15], so the impact of fear may not be limited to noise from aircraft, railways, and road traffic but also extend to other potentially hazardous noise sources, such as substation noise. As the urban acoustic environment in China becomes increasingly complicated, fear may be widely distributed across various living environments (e.g., neighborhoods and industrial zones), but its specific manifestations still require systematic research support.

#### 3.2.2. The Impact of Demographic Factors on Noise Annoyance

Unlike attitudinal factors, the impact of most demographic factors is controversial. Miedema and Vos also suggested that the importance of demographic factors is much less than that of attitudinal factors [20]. Different studies have collected varying information depending on the research questions. Overall, gender and age are the most frequently explored factors, and the specific research status of each factor is as follows.

The impact of gender on noise annoyance is controversial and may be moderated by various factors, such as daily activity patterns, developmental stages, and noise types. Just like Miedema’s research, the majority of studies (13 in total) in China support the view that gender is not a significant influencing factor. However, nine studies did report the impact of gender on noise annoyance, though the conclusions are inconsistent (Table 3). Some studies suggest that women exhibit higher annoyance levels in certain situations [32,33,39,40,41,42], while others found that men experience more annoyance [37,38,43]. Research indicating that men experience more annoyance shows some peculiarities compared with female groups. For instance, the research method of tracking daily activities may be influenced by gender differences in daily activity patterns, while studies focused on children have certain peculiarities due to their developmental stages. Further evidence suggests gender differences in the perception of certain types of noise. Cai et al. found that when using water sound masking, relief from annoyance was greater for female participants than for male participants [40]. Even under the influence of water sound masking, some studies have still confirmed higher noise annoyance in females [32,41]. In light of these insights, the validity of these effects and their specific mechanisms are worthy of further investigation and in-depth analysis.

Age is a significant factor influencing noise annoyance, with varying effects observed across different studies. In most cases, noise annoyance is positively correlated with age [39,43,44,45,46]. Wei et al. found that the day–night threshold for middle-aged and elderly individuals was 3–5 dB higher than that of younger individuals [47], which may be related to the more severe health issues faced by older adults [30]. Higher levels of noise annoyance can sometimes be observed in specific age groups, as confirmed by Ni et al. [48] and Zheng et al. [26]. Additionally, both of Zhang et al.’s studies [37,38] identified an impact of age on children’s perception of noise annoyance, despite reaching different conclusions, suggesting that even a small age range can lead to significant variations in noise annoyance levels. Ni et al. [48] conducted a summary of related studies, but the limited number of studies presents certain limitations, and their analysis mainly focused on specific LAeq without fully considering the effects of noise intensity. Compared with Miedema’s research, a quantitative analysis needs to be conducted to observe the effect of age on the Chinese community response to noise. Additionally, more empirical studies are needed to better understand the relationship between age and noise annoyance. Furthermore, differences in physiological and psychological development across age stages may lead to distinct perceptions of noise, and whether children show unique noise annoyance reactions remains an area for further investigation.

The history of noise exposure can reduce individuals’ levels of annoyance. For Zhang et al. [35], a higher LAeq,48h experienced by participants was linked to lower noise annoyance, possibly because prolonged exposure to high noise levels increases the participants’ adaptability to noise [32]. Similarly, Wang et al. found that office staff in buildings near subway stations reported lower annoyance levels than non-office staff [33], and Qu et al. found that residents with a longer duration of residence were less likely to experience annoyance [39]. However, adaptation to specific noise types does not diminish annoyance responses to other noise sources. According to Di et al. [45], individuals working in high-noise environments were more likely to be annoyed by substation noise heard at home. Therefore, in actual research, matching noise exposure history with the target noise type is important to avoid confounding effects from different noise sources.

Two studies introduced the variable of daily headphone use frequency. For Wang et al. [49], lower headphone use frequency was associated with higher perceived noise annoyance, while Peng et al. found that individuals with lower headphone use frequency were more likely to experience reduced annoyance under the effect of masking noise [50]. However, this may be due to the long-term effects of headphone use on hearing rather than noise exposure history.

Similar to Miedema’s research, the use of noise sources, such as traveling by car or plane, can also relieve individuals’ perceptions of annoyance. For Li et al. [51], Qu et al. [39], Zhang et al. [44], and Chan et al. [34], individuals who frequently travel by car or plane report lower levels of noise annoyance. On the one hand, as mentioned earlier, frequent exposure enhances an individual’s adaptability to noise. On the other hand, this reflects a certain dependence on the noise source, which reduces an individual’s negative attitude toward the source [20], thereby reducing their annoyance level. Furthermore, Li et al. [51] also found that the purpose for traveling by subway has a similar effect: subway passengers traveling to work report lower noise annoyance than others, further confirming the moderating role of dependency on the noise source in noise annoyance.

Regarding education level and income, these two factors not only directly affect the level of annoyance but also may indirectly influence the perception of annoyance through other factors. In most cases, individuals with higher levels of education tend to report higher levels of noise annoyance [22,23,27,28], while Qu found that those with an education level below high school are more annoyed by aircraft noise [39]. Similarly, both high- [35,45] and low-income groups [39,43] have exhibited higher levels of annoyance in different studies. As observed by synthesizing the aforementioned studies (Table 4), when research involves multiple regions, individuals with lower education or income levels tend to exhibit higher noise annoyance. However, when the study area is fixed or the study is conducted in laboratory settings, individuals with higher levels of education or income are more likely to experience greater annoyance. This trend is consistent with the hypothesis put forward by Cai et al. [43], which posits that individuals with lower levels of education and income tend to reside in areas with higher levels of noise pollution, while those with higher education or income are more likely to exhibit heightened concerns about the impact of noise. Regrettably, due to the lack of research on housing-related factors, we cannot determine the accuracy of the abovementioned hypothesis. What can be confirmed is that the potential impact of study area delineation should be carefully considered to avoid biases caused by regional differences.

Even within the same environment, ownership of a house can influence the perception of annoyance. Compared with renters, homeowners are more likely to experience annoyance [29,39,52]. Chen et al. suggest that renters typically have lower income levels and are therefore more accustomed to a lower quality of life [52], while homeowners may be more sensitive to the impact of noise.

People in poor health are more likely to experience annoyance [42,46,47,53,54]. Li et al. found that individuals who consider their health status to be bad have a 1.38-times higher probability of experiencing annoyance compared with others (*p* < 0.001) [46]. Of note, the causal relationship between noise annoyance and health status remains unclear. Qu’s research confirmed that higher levels of noise annoyance are associated with a greater likelihood of poor health [39], suggesting that noise annoyance may not only be influenced by health status but also have a negative impact on health.

An individual’s marital status (i.e., married or other) has no impact on their perception of annoyance [35,46]. However, Cai et al. further subdivided marital status into married, unmarried, divorced, and widowed and found that both the married and unmarried groups experience higher levels of annoyance compared with that of the other two groups [43]. This difference may be due to the characteristics of the divorced and widowed groups being overshadowed by those of the unmarried group within the ‘other’ category. Therefore, to gain a deeper understanding of the impact of marital status on noise annoyance perception, a more detailed classification and study of different marital statuses is necessary.

Perception of noise annoyance can be influenced by both employment status [29] and type of occupation [44]. According to studies by Wang [53] and Wei [55], knowledge workers exhibit higher susceptibility to annoyance compared with manual workers, potentially attributable to their heightened demands for acoustic environmental quality. However, some studies have shown that occupation has no impact on noise annoyance [35,47].

#### 3.2.3. The Impact of Situational Factors on Noise Annoyance

Situational factors are relatively limited, with only three known aspects: visibility of the noise source, time spent at home, and time at home.

The impact of noise source visibility has yielded different conclusions. According to Zhang et al. [56], noise annoyance was significantly higher when the noise source was visible compared with when it was not. However, in a laboratory study, Sun et al. found that noise annoyance was relatively lower when the noise source was visible, and this effect was greater than that of green visual elements [57]. This discrepancy may stem from differences in the used research methods. A laboratory-based study [57] suggested that when visual information aligns with an individual’s noise perception, it can somewhat reduce their levels of annoyance. In contrast, field-based studies involving participants who have long-term exposure to a specific noise environment showed adaptation to the visibility of the noise source. Therefore, it is essential to consider that changes in visual elements can influence noise annoyance, and the visibility of noise sources is often closely linked to the overall visual landscape. For example, in the three aforementioned studies, reducing the visibility of noise sources was accompanied by interventions of other visual elements. The presence of a noise source itself can also affect the visual landscape, as demonstrated by Song et al. [30], who found that the negative visual impact caused by wind turbines exacerbates noise annoyance. Therefore, as discussed by Sun [57], the interplay between noise source visibility and visual elements requires further investigation to achieve a more comprehensive understanding of the complex relationship between auditory and visual factors in noise perception.

Additionally, home-stay patterns have an impact: individuals who spend less time at home tend to experience higher noise annoyance [46], and daily home-stay periods can influence annoyance levels [58]. Of note, different home-stay patterns may correspond to different population groups, for example, working individuals spend less time at home, while students are primarily at home on weekends. A more in-depth analysis involving multiple factors should be conducted, and the specific mechanisms underlying this effect require further investigation.

## 4. Discussion

As the most important part of this study, based on the results of the analysis on the impact of non-acoustic factors on the Chinese community response to noise, the factors explored in this study can be presented at three levels. The first level is the factors that have a relatively consistent impact on noise annoyance: noise sensitivity, attitude to noise source, health status, perceived quality of the living environment, and education level. Based on a certain number of studies, these factors show a relatively consistent trend of influence on noise annoyance. The second level is the factors that have controversial impacts on noise annoyance: gender, age, and occupation. These factors exhibit different influences on noise annoyance in various studies, and discerning their impact on the group’s response through a qualitative analysis is difficult. The third level is the factors that have been paid insufficient attention, that is, other factors besides the abovementioned ones. Some of these factors have been quantitatively analyzed in Miedema’s research, and fear, in particular, has been proven to have an impact equivalent to 19 dB DNL. Therefore, research needs to be conducted on the influence of these factors.

The factors at the first level all exhibit the same trends as those in international studies. As for the unique influences of non-acoustic factors within the Chinese population, a quantitative analysis is needed to explore these. On the one hand, we can compare the differences in the effect sizes of these factors. On the other hand, we can specifically understand the influence of the factors at the second level. Taking age as an example, although most studies show influencing trends different from those in Miedema’s summary, a quantitative analysis integrating data from all relevant studies is still needed to assess age’s influence from an overall research perspective.

In addition to the need for a quantitative analysis, due to the limited number of local Chinese studies, the following key issues still need to be addressed:

(1) Exploration of interactions between certain factors

Some of the reviewed studies have conducted preliminary explorations of the interactions between certain factors, such as age and health status [30], age and gender [37,38], and noise source visibility and noise sensitivity [57], which offer valuable insights. Furthermore, various other interactions, such as gender and noise sensitivity or age and time spent at home, may be critical for understanding the mechanisms through which non-acoustic factors influence noise annoyance. However, due to the limited research data, further in-depth investigations in this area remain challenging.

(2) Interaction between non-acoustic factors and noise types

Different exposure–response relationships across various noise types have been extensively studied [59,60,61]. The differences in the influence of non-acoustic factors across various noise types have also been reflected in the reviewed studies. For instance, Song et al. [27] found that the annoyance level differences between high- and low-sensitivity individuals varied between speech and road traffic noise. Similarly, Lam et al. [28] reported differences in the impact pathways of various factors on annoyance caused by road and railway noise. Exploring the interaction between non-acoustic factors and noise types is therefore essential for understanding the mechanisms underlying non-acoustic influences and for developing noise policies that are more aligned with real-world conditions.

In terms of research methods, 26 studies explored the factors influencing long-term annoyance through social surveys and 15 studies focused on short-term annoyance through laboratory experiments. Moreover, one study carried out research through a literature review. Due to the limited number of studies, this study did not conduct separate analyses of long- and short-term annoyance. As for the analysis methods, they are closely related to the research objectives of each piece of literature. Looking back at Figure 2, most studies took non-acoustic factors as one of the factors for predicting annoyance level rather than specifically studying the effect of non-acoustic factors. Therefore, the logistic regression method was mostly adopted to test the role of each factor, making obtaining specific effect values for the influence of each factor difficult. However, the data accumulated from these studies are of great significance for future quantitative analyses.

Overall, research on the impact of non-acoustic factors on noise annoyance in China remains relatively limited. In the future, more high-quality local Chinese studies are needed to establish a solid foundation for in-depth analyses, such as the quantification of impact effects, and to develop a noise assessment framework tailored to local socio-cultural characteristics, providing a precise scientific basis for environmental noise management and policy-making.

## 5. Conclusions

This study provides the first systematic review of research on non-acoustic factors in China and their influence on noise annoyance among Chinese residents. To achieve this, a systematic review of four major scientific databases was conducted, followed by a qualitative analysis of the selected studies. The main conclusions are as follows:

(1) The effects of non-acoustic factors on Chinese residents are significant, with the trends for factors such as noise sensitivity, attitude to noise source, health status, perceived quality of the living environment, and education level influencing the Chinese community response to noise having been basically clarified. However, the influence of the remaining factors and the unique influences of various non-acoustic factors await further quantitative analysis.
(2) Interactions among various factors deserve close attention. The interactions between non-acoustic factors, as well as those between non-acoustic factors and sound source types, have been reflected in some studies. These may be significant for explaining the effects of non-acoustic factors and merit further research.
(3) Compared to international studies, research on non-acoustic factors in China is relatively limited in quantity and unevenly distributed, which is insufficient to support further quantitative analysis or a detailed exploration of the underlying mechanisms. Therefore, more studies are necessary to support the future rationalization of noise policies and national standards in China.

## Figures and Tables

**Table 1:** Non-acoustic factors and their categorization.

Attitudinal Factors	Demographic Factors	Situation Factors
**Noise sensitivity (20)**	**Gender (23)**	Visibility of the source (3)
**Attitude to noise source (6)**	Age * (20)	Time spent at home (1)
**Perceived quality of the** **living environment (5)**	**Education level (7)**	Time at home (1)
Activity during exposure (3)	**Occupation (7)**	*Sound proofing*
Fear (1)	**Health condition (6)**	*Dwelling orientation*
*Health effect*	Use of the noise source (4)	
	Income (4)	
	Marital situation (3)	
	Ownership of a house (3)	
	History of noise exposure (3)	
	Length of residence (1)	
	Dependency on the noise source (1)	
	*No. of people in the household*	
	*Social status*	
	*Size of household*	
	*Type of home*	

Note: factors in bold denote those examined in five or more studies, italicized text indicates factors that appeared frequently in international summaries but were not covered in the Chinese studies, the number in the bracket indicates the quantity of studies that involve the factor, and * indicates that the impact of the factor on the Chinese community response to annoyance has been summarized or analyzed.

**Table 2:** The definitions of attitude to noise sources used in the reviewed research.

Research	Definition of Attitude to Noise Source	Categories
Lam et al., 2008 [31] AND Lam et al., 2009 [28]	Comparing railway and road traffic, do you agree that railway/road traffic is comfortable AND convenient AND environmentally friendly AND noisy AND safe	Comprehensive attitude
Song et al., 2016 [30]	Attitudes towards wind turbines’ visual impact on the landscape AND general opinions on wind turbines	
Zhang et al., 2021 [29]	Considering high-speed railway safer OR expressing more support for high-speed rail construction	
Wang et al., 2022 [33]	Metro noise attitude: 1 as “not at all”, 5 as “extremely noisy”	Single impact
Wang et al., 2022 [32]	Do you agree that you are a person who strongly hates metro noise?	

**Table 3:** Studies reporting gender differences.

Research	Type of Noise	Conclusions	Notes
Leung et al., 2017 [41]	Water sounds and road traffic noise	Females experience higher levels of annoyance	\
Wang et al., 2022 [32]	Water sounds and metro noise		\
Qu et al., 2023 [39]	Aircraft noise		\
Yan et al., 2009 [42]	Road traffic noise		\
Wang et al., 2022 [33]	Metro noise		\
Cai et al., 2023 [43]	Environmental noise	Males experience higher levels of annoyance	Tracking daily activities
Zhang et al., 2022 [38]	Road traffic noise, white noise, low-frequency noise		Focus on children
Zhang et al., 2018 [37]	Road traffic noise, white noise, air conditioner noise		Focus on children
Cai et al., 2019 [40]	Water sounds and industrial noise	Relief from annoyance was greater for female participants	\

**Table 4:** Study areas in research on education level and income.

Factor	Research	Study Area	Conclusions
Education level	Qu et al., 2023 [39]	Three communities	Individuals with lower education levels tend to experience higher levels of annoyance
	Di et al., 2022 [45]	Laboratory experiment	Individuals with higher education levels tend to experience higher levels of annoyance
	Li et al., 2012 [46]	Single community	
	Zhang et al., 2020 [35]	Single community	
	Chen et al., 2007 [52]	Four different sites	
Income	Qu et al., 2023 [39]	Three communities	Low-income groups tend to experience higher levels of annoyance
	Cai et al., 2023 [43]	Two communities with different environments	
	Di et al., 2022 [45]	Laboratory experiment	High-income groups tend to experience higher levels of annoyance
	Zhang et al., 2020 [35]	Single community	

## Data Availability

All data were sourced from published studies. Methodological details and processed datasets are available from the corresponding author upon request.
